# Measuring and Modeling the Effect of Surface Moisture on the Spectral Reflectance of Coastal Beach Sand

**DOI:** 10.1371/journal.pone.0112151

**Published:** 2014-11-10

**Authors:** Corjan Nolet, Ate Poortinga, Peter Roosjen, Harm Bartholomeus, Gerben Ruessink

**Affiliations:** 1 Soil Physics and Land Management, Wageningen University, Wageningen, The Netherlands; 2 Laboratory of Geo-information Science and Remote Sensing, Wageningen University, Wageningen, The Netherlands; 3 Department of Physical Geography, Faculty of Geosciences, Utrecht University, Utrecht, The Netherlands; Centro de Investigacion Cientifica y Educacion Superior de Ensenada, Mexico

## Abstract

Surface moisture is an important supply limiting factor for aeolian sand transport, which is the primary driver of coastal dune development. As such, it is critical to account for the control of surface moisture on available sand for dune building. Optical remote sensing has the potential to measure surface moisture at a high spatio-temporal resolution. It is based on the principle that wet sand appears darker than dry sand: it is less reflective. The goals of this study are (1) to measure and model reflectance under controlled laboratory conditions as function of wavelength (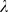) and surface moisture (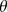) over the optical domain of 350–2500 nm, and (2) to explore the implications of our laboratory findings for accurately mapping the distribution of surface moisture under natural conditions. A laboratory spectroscopy experiment was conducted to measure spectral reflectance (1 nm interval) under different surface moisture conditions using beach sand. A non-linear increase of reflectance upon drying was observed over the full range of wavelengths. Two models were developed and tested. The first model is grounded in optics and describes the proportional contribution of scattering and absorption of light by pore water in an unsaturated sand matrix. The second model is grounded in soil physics and links the hydraulic behaviour of pore water in an unsaturated sand matrix to its optical properties. The optical model performed well for volumetric moisture content 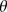 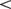 24% (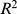 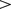 0.97), but underestimated reflectance for 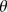 between 24–30% (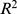 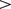 0.92), most notable around the 1940 nm water absorption peak. The soil-physical model performed very well (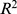 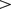 0.99) but is limited to 4% 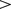 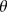 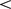 24%. Results from a field experiment show that a short-wave infrared terrestrial laser scanner (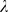 = 1550 nm) can accurately relate surface moisture to reflectance (standard error 2.6%), demonstrating its potential to derive spatially extensive surface moisture maps of a natural coastal beach.

## Introduction

Every decade sea water level of the North Sea rises by 2–3 cm [1]. This is alarming for a country as the Netherlands considering large parts are already below sea level. It puts urgency on finding coastal defense strategies that are able to adapt to climate change [2]–[4]. In this context an unprecedented large nourishment of sand (‘Sand Motor’, www.zandmotor.nl) was laid down along a stretch of the Dutch coast in 2011. Its aim is to mimic the onshore migration of an intertidal sandbar, supplying the adjacent coast with a surplus of sand for years to come [5], [6]. An important expected result is the transport of sand by wind over the beach towards the dunes, enabling the dunes to naturally grow in volume [7], [8].

Surface moisture is an important supply limiting factor for aeolian sand transport [9]–[14]. By binding sand grains together, through cohesive and adhesive forces, water significantly increases the resistance of the uppermost sand layer against wind erosion [15]–[17]. It has been suggested by [18] that the required wind force to initiate saltation grows exponentially with a linear increase of moisture content. Above a certain moisture content beach sand becomes inherently resistant to entrainment by most natural winds.

To accurately predict aeolian sand availability for dune building it is thus critical to account for the control of surface moisture. However, wetting and drying processes are governed by complex hydraulics of tidal and wave action, groundwater and capillary flow, and evaporation and precipitation [19]–[23]. As a result, the distribution of surface moisture on a beach can vary greatly in space and time. Therefore, to estimate the control surface moisture exerts on aeolian sand transport, data at a high spatio-temporal resolution is required [24]–[26].

Optical remote sensing can be a viable solution for measuring surface moisture at a high spatio-temporal resolution. It is based on the principle that wet sand appears darker than dry sand: it is less reflective. This familiar reduction in reflectance is attributed to pore water surrounding the sand grains, causing a change in scattering and absorption of light. Scattering and absorption of sunlight occur at the same time, but their proportional contribution to reduction in reflectance depends on wavelength (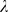) and moisture content (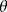) [27]–[29]. This holds true when other parameters affecting beach surface reflectance (e.g. mineral composition, grain size distribution, packing density, surface roughness [30], [31]) remain unchanged.

The potential of optical remote sensing of surface moisture, for aeolian research in the coastal environment, was first demonstrated by [32]–[35] and [36]. Through a photographic methodology (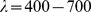 nm), beach surface moisture content was related to a corresponding normalized surface reflectance. This relationship was applied to photographs of a beach, resulting in a time-series of surface moisture maps. The same principle was later tested by [37], [38] and [39], where the reflective signal of a terrestrial laser scanner (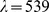 nm) was related to beach surface moisture. It is a more convenient application since the reflected signal does not require a correction for changes in illumination. Another application of the principle was tested by [40] and [41], where point data on beach surface moisture was collected using a portable narrow band radiometer (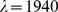 nm) and spectroradiometer (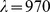 nm). At these wavelengths the correlation between surface reflectance and moisture content was shown to be higher than at visible wavelengths, due to stronger absorption of light in water.

**Figure 1:**
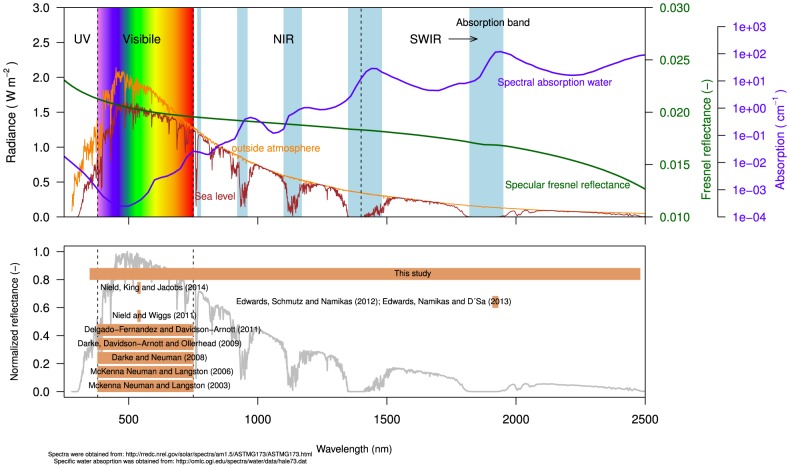
Spectral power distribution of sunlight (in Wm^−2^) outside the atmosphere (yellow line) and at sea level (brown line), to illustrate the effect of absorption of sunlight by water. Included in the top panel: spectral Fresnel reflectance (-) for a water surface (green line) and absorption coefficients (in cm^−1^) for pure water (blue line). Bottom panel: overview of the studies (including this study) that demonstrate the potential of relating reflectance to surface moisture. The orange bars indicate the spectral range in which the measurements were taken.

In soil science, optical remote sensing is widely used to determine soil moisture content [27], [28], [42]–[44]. However, the recent studies that relate reflectance to surface moisture in a coastal environment, show ambiguous results, as was also recognized by [40] and [41]. It is in part due to the focus on a limited range of wavelengths in which measurements were taken (see overview in Figure 1-bottom). The goals of this study are (1) to measure and model reflectance under controlled laboratory conditions as function of wavelength (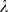) and surface moisture content (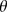) over the full optical domain of 350–2500 nm, and (2) to explore the implications of our laboratory findings for accurately mapping the distribution of surface moisture under natural conditions. A laboratory spectroscopy experiment is conducted to measure spectral reflectance (1 nm interval) under different surface moisture conditions using beach sand. Two models are developed and tested. The first model is grounded in optics and describes the proportional contribution of scattering and absorption of light by pore water in an unsaturated sand matrix. The second model is grounded in soil physics and links the hydraulic behaviour of pore water in an unsaturated sand matrix to its optical properties. A field experiment is conducted to test the potential of a short-wave infrared terrestrial laser scanner (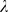 = 1550 nm) to derive spatially extensive surface moisture maps with a high accuracy. As such, this study aims to support practical applications for optical remote sensing of surface moisture on a sandy coastal beach.

## Background

### Spectral reflectance

Elastic scattering of light is the re-directing of light by a medium without alteration of the wavelength. It encompasses the optical phenomena of reflection, refraction, and diffraction [45]–[47] which are determined by wavelength, angle of incidence and optical properties of the medium. Transmission of sunlight into opaque beach sand is in the order of a few sand grains thick [30], [31], [48], [49]. Optical reflectance is thus strictly a surface phenomenon.

With optics Ängstrom [50] proposed an explanation for the familiar visual darkening of sand upon wetting, ascribing it to total internal reflection within water films surrounding the sand grains. In effect, sunlight at or exceeding the critical angle is reflected back to the surface at the liquid-air interface, increasing the likelihood of being absorbed by the sand grains. Assuming ideal diffuse reflection [50] described the relation between wet (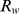) and dry (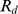) reflectance by:
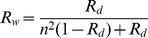
(1)With 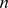 the refractive index of water. Lekner and Dorf [51] later modified this model to account for the fraction of sunlight that is not transmitted into the water film but reflects specularly (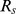) at the air-liquid interface:
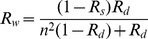
(2)




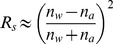
(3)Specular reflectance 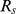 is approximated by Fresnel reflection of normal incident sunlight [45], with 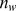 the refractive index of water and 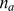 the refractive index of air, taken as 1. How specular reflectance changes with wavelength is shown in Figure 1.

The process of absorption is also important to spectral reflectance. Absorption of sunlight is the uptake of light by conversion of its energy into thermal energy. It is strongly correlated to wavelength. Spectral absorption of sunlight is described by the Beer-Lambert law [46], stating an exponential decrease of reflectance as a function of the absorption coefficient 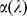 (cm^−1^) and optical path length 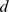 (cm):
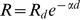
(4)


The spectral absorption coefficient describes the extent to which sunlight is absorbed as it passes through a medium. Figure 1 shows the absorption coefficients for pure water in the optical domain 350–2500 nm (data: [52]). It becomes clear from Figure 1 that water is a strong absorber of sunlight at specific wavelengths. Notable absorption peaks all occur at near- and shortwave-infrared wavelengths, around 760, 970, 1200, 1470 and 1940 nm [52], [53]. At visible wavelengths the absorption coefficient is close to zero. Here, the penetration depth (i.e. inverse of absorption) of sunlight in water is high, which is why water appears transparent.

The effect of absorption of sunlight by water is illustrated by comparing the spectral power distribution (SPD) of sunlight (Wm^−2^) outside the atmosphere to that at sea level (Figure 1). Since water (vapour) is abundant in the atmosphere, certain wavelengths of sunlight are absorbed to such an extent that it may not reach the earth's surface. This is of consequence for collecting remotely sensed data. Passive methods, that depend on sunlight for acquiring information about an object, are ‘short-sighted’ or in fact blind in wavelengths strongly absorbed by water. This is true for wavelengths around the 1470 and 1940 nm absorption peaks. In these wavebands an active method must be employed, where data is collected using a light source other than the sun [54].

### Surface moisture

**Figure 2:**
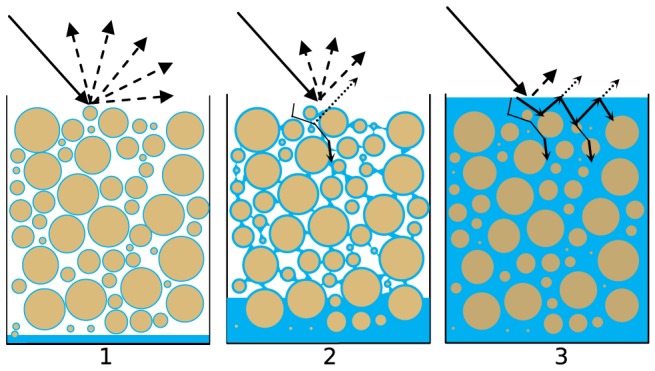
Conceptual representation of the non-linear decrease of spectral reflectance as moisture content in the sand matrix increases. At wilting point (1) there is almost no absorption of light in water as the optical path length (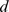) is close to zero. At field capacity (2) the optical path length in water (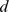) increases with increasing absorption as result. At saturation (3) the optical path length in water (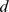) is at its maximum and certain wavelengths may be completely absorbed.

The decrease of spectral reflectance upon wetting is non-linear, as is the hydraulic behaviour of water in an unsaturated sand matrix. Both processes can be linked conceptually, as is shown in Figure 2. At (and below) wilting point (Fig. 2.1) pore water is held tightly in the sand matrix as adsorbed water films around the sand grains. Here the optical path length in water (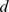) is close to zero, and the decrease of spectral reflectance is almost solely due to scattering. Approaching field capacity (Fig. 2.2) pore water proceeds to fill micro pores and form water wedges between sand grains. This increases the optical path length in water (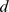), with increasing absorption as result. When the sand matrix gets fully saturated (Fig. 2.3) all remaining air in the sand matrix is replaced with water and free water may appear at the surface. Now the optical path length in water (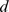) is at its maximum and certain wavelengths may be completely absorbed [28], [55].

**Figure 3:**
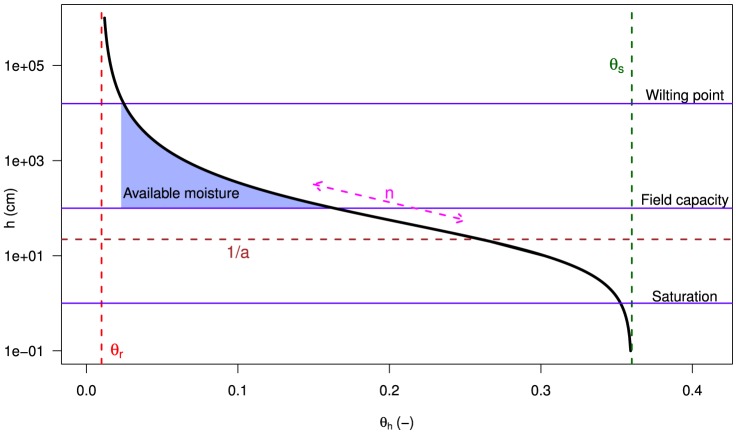
Water retention curve for a coarse grained sand matrix (M50 = 350–500 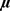
m), created using the Van Genuchten equation (Eq. 5). Shown in the graph are the residual water content (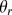), saturated water content (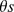), air entry value (1/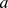) at the inflection point, and (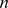) which is related to the slope at inflection point and indicates pore-size distribution. Data taken from [63].

The hydraulic behaviour of water in an unsaturated sand matrix is described by the water retention curve (Fig. 3), relating volumetric water content 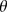 to the water pressure head 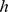 in cm. Under unsaturated conditions the water pressure head is always negative, for cohesive and adhesive forces in the sand matrix reduce pore water potential relative to free water. A well-established empirical model to describe the water retention curve is the Van Genuchten equation (Eq. 5) of [56]:
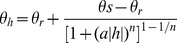
(5)


With 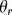 the residual water content and 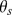 the saturated water content. At these water contents the gradient 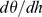 of the water retention curve becomes zero. Parameter 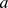 (cm^−1^) approximately equals the inverse of the pressure head at the inflection point, where 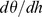 has its maximum value. It is interpreted as the air entry value. The dimensionless parameter 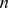 relates to the slope at the inflection point, thus reflecting steepness of the water retention curve. It is is interpreted as an indicator of pore-size distribution [56], [57]. Note that moisture levels of 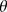 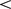 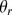 and 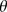 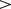 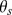 are beyond the range of Eq. 5.

### Surface moisture - spectral reflectance models

Two simple models are proposed to obtain a description of spectral reflectance under different moisture conditions. The first model (Eq. 6) is grounded in optics. Spectral reflectance 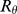 as a function of volumetric surface moisture is described by:
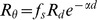
(6)


Where parameters 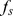 (fraction) and 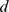 (cm) at a certain wavelength and moisture content, are obtained by curve fitting. Parameter 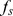 describes the contribution of elastic scattering to reduction of spectral reflectance upon wetting, while parameter 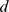 (optical path length in water) describes the contribution of absorption to reduction of spectral reflectance upon wetting. Further, 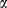 denotes the wavelength dependent absorption coefficient for pure water, and 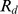 the dry spectral reflectance. The model is based on the approach of [58], with omission of the fraction 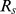 as the contribution of specular reflectance to darkening upon wetting was shown by [58] to be minimal. The model of [58] is similar to the model of [28], although the latter model takes the absorption coefficient 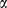 as a regression parameter.

The second model (Eq. 7) is grounded in soil physics. The Van Genuchten equation (Eq. 5) is modified by replacing pressure head 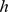 (cm) with spectral reflectance 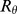. Parameters 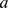 and 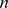, at a certain wavelength, are obtained by curve fitting and become dimensionless as spectral reflectance is a fraction. Volumetric moisture content 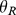 as a function of spectral reflectance is described by:
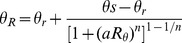
(7)Where residual water content 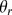 and the saturated water content 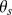 are sand matrix constants. Note that the soil-physical model is fitted at a certain wavelength, whereas the optical model is fitted over all wavelengths in the optical domain.

## Materials and Methods

### Experimental setup

A laboratory spectroscopy experiment was conducted in duplo to observe spectral reflectance in the optical domain (350–2500 nm) under different moisture conditions. A representative sample of beach sand was collected from the ‘Sand Motor’ (GPS location: 52.052°N 4.184°E). A field permit was not required and sample collection did not involve endangered or protected species of flora or fauna. Before the experiment the sample was coarsely sieved (2 mm) to remove shells and constituents other than sand. The sand, composed of quartz with some feldspar, had a dry bulk density 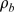 of 1.655 gcm^−3^ with mean and median grain size of 324 and 288 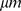 respectively.

For each experiment, a sub-sample of the collected beach sand was placed in a matte black petridish (5 cm radius, 1.5 cm height), filling it up to the rim, and oven dried for 24h at 105 °C. The sample was, after measuring its initial weight, slowly saturated with distilled water. The water was let to distribute itself uniformly in the sample and excess free water was drained from the surface. The sample was placed on a data-logging weighing scale with milligram precision.

During the drying process the reflectance (correct terminology: biconical reflectance factors or BCRF's) of the sample were acquired using the spectroscopy facility of Wageningen University [59]. The spectral reflectance was measured at 1 nm intervals using an ASD Fieldspec Pro spectrometer (Analytical Spectral Devices, Boulder, CO). A 40×40 cm white Spectralon panel (LabSphere, Inc., North Sutton, NH) was used to calibrate the spectrometer.

**Figure 4:**
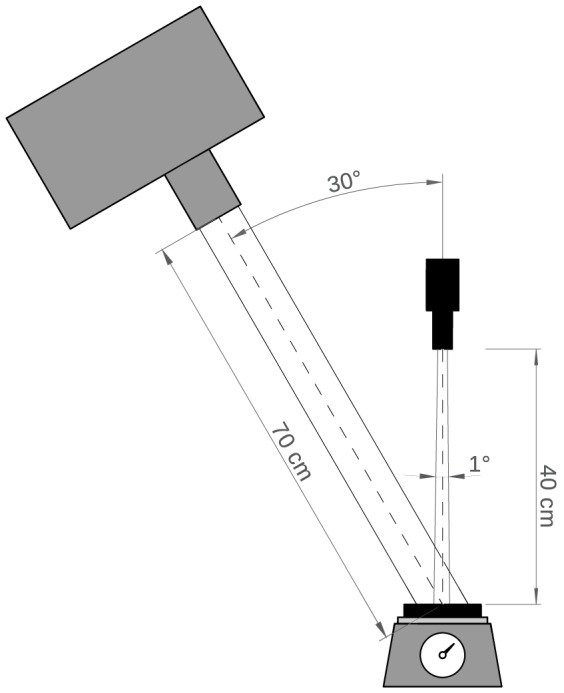
Measurement setup of the laboratory spectroscopy experiment. An ASD Fieldspec Pro spectrometer (Analytical Spectral Devices, Boulder, CO), fitted with a 1° FOV foreoptic, was directed at nadir at 40 cm distance from the sample. A 900 watt Quartz Tungsten Halogen (QTH) lamp was placed 70 cm from the sample at a 30° zenith angle. The spectral reflectance was measured over a range of 350–2500 nm, at 1 nm intervals.

The spectrometer was fitted with a 1° FOV foreoptic which was directed at nadir at 40 cm distance from the sample. As an artificial light source, a 900 watt Quartz Tungsten Halogen (QTH) lamp was placed 70 cm from the sample at a 30° zenith angle (see Figure 4). At the time of the measurements the room temperature was kept stable at 23 °C and the humidity was kept constant at 50%. The spectrometer was programmed to take a measurement every 5 minutes. At the same time the weight of the sample was measured and stored. Data are publicly accessible at doi:10.4121/uuid:866135c2-2be3-4b74-8f9c-922505285a7b.

### Analysis method

For each sub-sample the laboratory spectroscopy experiment obtained five-minute interval measurements of spectral reflectance at a certain volumetric moisture content. Only wavelengths between 350–2100 nm were included in the analysis, as wavelengths 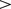 2100 nm were found to have a lower signal-to-noise ratio. Volumetric moisture content was calculated by:
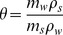
(8)With 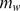 and 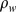 the (decreasing) mass and density (0.997 gcm^−3^) of water, and 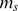 and 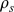 the mass and dry bulk density (1.655 gcm^−3^) of the sand sample.

Equation 6 and 7 were fitted to the spectral reflectance measurements. Parameters 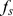 and 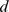 (Eq. 6) and 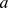 and 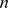 (Eq. 7) were optimized using a non-linear (weighted) least-squares regression algorithm [60]. The spectrum of the air-dry sample was used for 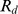 and the spectrum for 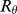 corresponded to a certain volumetric moisture content. The sand matrix constants 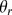 and 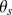 were determined manually, using the graphical relationship between spectral reflectance and volumetric moisture content.

## Results and Discussion

### Laboratory experiment

The laboratory spectroscopy experiment was conducted in duplo to assess the variation in spectral reflectance of the beach sand as function of surface moisture content. The spectral reflectance curves of both experiments (interpolated to regular moisture intervals) showed minimal variation between 400–2100 nm (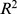 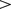 0.997). Therefore the dataset with most data points was selected for further analysis. A total of 300 spectral reflectance measurements were taken over a period of 25 hours. Volumetric surface moisture content varied between 32% (saturation) and 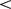 0.01% (air-dry).

**Figure 5:**
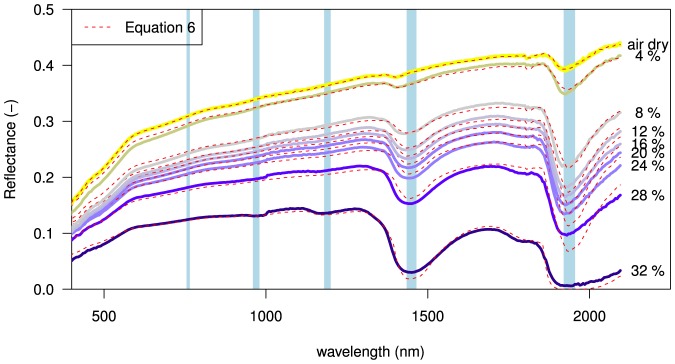
Measured spectral reflectance over a range of 350–2100 nm at 4% volumetric moisture content interval, between 32% (saturation) and 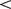 0.01% (air-dry). A non-linear decrease in reflectance upon wetting is observed over the full range of wavelengths. Notable dips in reflectance occur at water absorption peaks at 1470 and 1940 nm (blue bands). The dashed red lines show the spectral reflectance 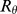 as calculated by fitting the optical model (Eq. 6) to measured spectral reflectance.

Figure 5 shows measured spectral reflectance plotted at 4% moisture intervals. A non-linear decrease of reflectance, as moisture content increases, is observed over the full range of wavelengths. The shape of the air-dry spectral reflectance curve (top line Fig. 5) reflects the optical properties of the beach sand itself. Overall, at longer wavelengths, dry beach sand becomes more reflective but wet beach becomes less reflective. This is due to stronger absorption of light in water at near- and short-wave infrared wavelengths (see Fig. 1). Notable dips in reflectance are observed at the absorption peaks of 1470 and 1940 nm. The overall shape of the curve at visible wavelengths (400–700 nm) does not change greatly with increasing moisture content. This corresponds to the notion that soils darken when wet but with little apparent color change.

**Figure 6:**
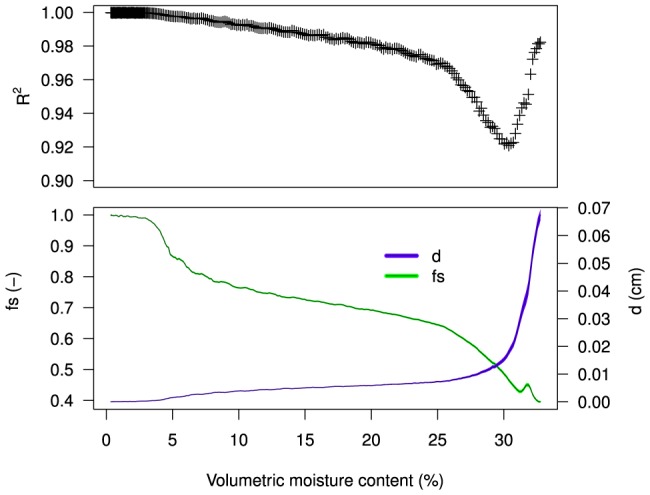
Performance of the optical model (Eq. 6). Top panel: goodness of fit 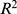 of the optical model as function of volumetric moisture content. Bottom panel: trajectories of the regression parameters 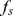 and 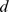 of the optical model, describing the proportional contribution of scattering (fraction 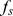) and absorption (optical path length in water 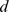) as moisture levels increase. Shaded areas indicate 95% confidence intervals.

The optical model (Eq. 6) was fitted to the spectral reflectance measurements and shown as dashed red lines in Figure 5. As can be seen in Figure 6 (top) the optical model performs well for surface moisture contents 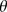 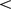 24% (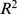 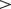 0.97), but gives an underestimation in reflectance for 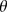 between 24–30% (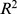 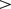 0.92). This is most notable around the 1940 nm water absorption peak (see Fig. 5). A plausible explanation is that the absorption of light is not as effective in pore water since it is partially bound to the sand matrix. This notion is supported by the fact that at saturation (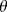 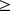 32%), where free water is present, model performance improves (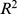 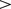 0.97).

Figure 6 (bottom) shows the values of 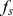 (fraction) and 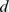 (cm) at corresponding moisture content. The trajectories of the regression parameters of the optical model describe the proportional contribution of elastic scattering (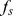) and water absorption (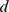) to spectral reflectance upon wetting. These trajectories are in agreement with the principle outlined in Figure 2. At air-dry conditions (Fig. 2.1) pore water is held tightly in the sand matrix. The optical path length in water (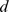) is close to zero as there is negligible absorption of light in water. Reflectance is thus almost solely due to scattering, and fraction 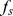 is close to 1. When pore water proceeds to fill micro pores and form water wedges between sand grains (Fig. 2.2), the contribution of absorption increases, while the contribution of scattering decreases. When the sand matrix gets fully saturated (Fig. 2.3) the optical path length in water (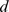) is at its maximum, between 0.06–0.07 cm. This corresponds to a thickness of a few sand grains (median 288 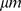). It is encouraging that the order of magnitude is within physical expectation.

**Figure 7:**
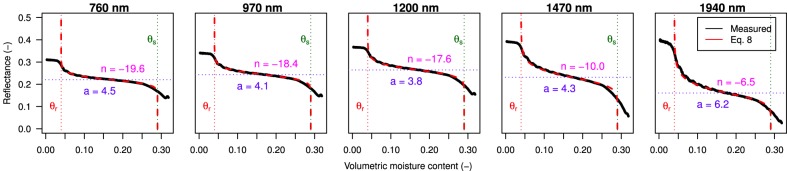
Measured spectral reflectance upon wetting at the water absorption peaks of 760, 970, 1200, 1470, and 1940 nm. The dashed red lines show volumetric moisture content 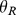 as calculated by fitting the soil-physical model (Eq. 7). With sand matrix parameters 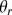 = 0.04 and 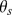 = 0.29. Non-linear regression parameters 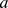 and 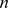 are shown in the plot at corresponding wavelength.

Figure 7 (black lines) shows measured reflectance as a function of volumetric moisture content plotted for the five water absorption peaks at 760, 970, 1200, 1470, and 1940 nm. It can be seen that at longer wavelengths the air-dry reflectance increases and reflectance upon wetting decreases, resulting in greater contrast between wet and dry reflectance. Wavelengths at 1470 and 1940 nm are absorbed by water to such an extent that saturated reflectance approaches zero. Further, it becomes clear from Figure 7 that the shape of the spectral reflectance curves are very similar to the shape of the water retention curve calculated by Eq. 5 of [56] (Fig. 3). This suggests that spectral reflectance influenced by surface moisture content is linked to the hydraulic behaviour of water in an unsaturated sand matrix. This link is conceivable, as both processes share common drivers such as mineral composition and texture of the soil. The spectral reflectance upon wetting and water retention characteristics of beach sand can thus be described by the same empirical formulation.

The soil-physical model (Eq. 7) was fitted to the reflectance curves of Figure 7. Non-linear regression parameters 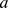 and 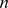 are shown in the plots at corresponding wavelength. The sand matrix parameters 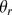 and 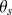 were determined at 0.04 and 0.29 respectively. As a consequence, moisture levels of 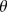 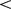 4% and 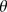 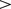 29% are beyond the range of the soil-physical model. While knowledge of 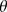 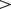 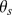 is not very relevant for aeolian coastal research, knowledge of 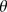 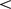 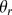 is relevant. Even at low levels, surface moisture exerts a significant control on the aeolian entrainment of sand. As can be seen in Figure 7, the reflectance for 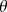 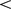 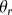 at shorter wavelengths remain almost level and a differentiation between moisture levels 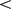 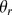 is not possible. At near- and short-wave infrared wavelengths, however, the reflectance does increase as moisture content decreases towards air-dry conditions. Here the approach of [61] offers an alternative to the soil physical model, as it can describe the reflectance curve over the full moisture range using cubical spline approximations.

**Figure 8:**
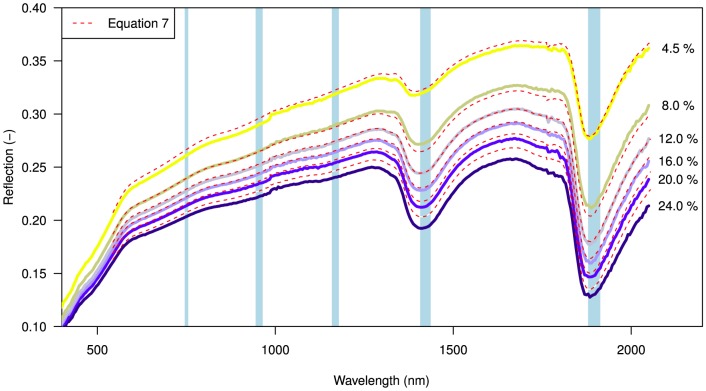
Spectral reflectance upon wetting (red dashed lines) obtained by fitting the soil-physical model (Eq.7) to measured spectral reflectance at 1 nm interval. The blue bands indicate spectral absorption peaks for water. Between 4.5–24% volumetric moisture content the spectral reflectance reconstructed with Eq. 7 has a goodness of fit of 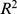 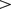 0.99.

The soil-physical model (Eq. 7) was also fitted to measured spectral reflectance upon wetting over the full range of 350–2100 nm, at 1 nm interval. By obtaining the values for 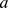 and 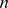 for all optical wavelengths for the unsaturated sand matrix between 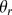 and 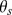, it was possible to calculate the spectral reflectance as a function of volumetric moisture content (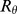). Figure 8 shows the calculated (red dashed lines) and measured (continuous lines) spectral reflectance upon wetting between 4.5–24% volumetric moisture content plotted at 4% moisture content interval. Between these moisture levels the reconstructed spectral reflectance has an overall goodness of fit of 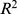 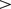 0.99. A slight underestimation in spectral reflectance was found for higher surface moisture contents, most notable around the absorption peaks of 1470 and 1940 nm.

**Figure 9:**
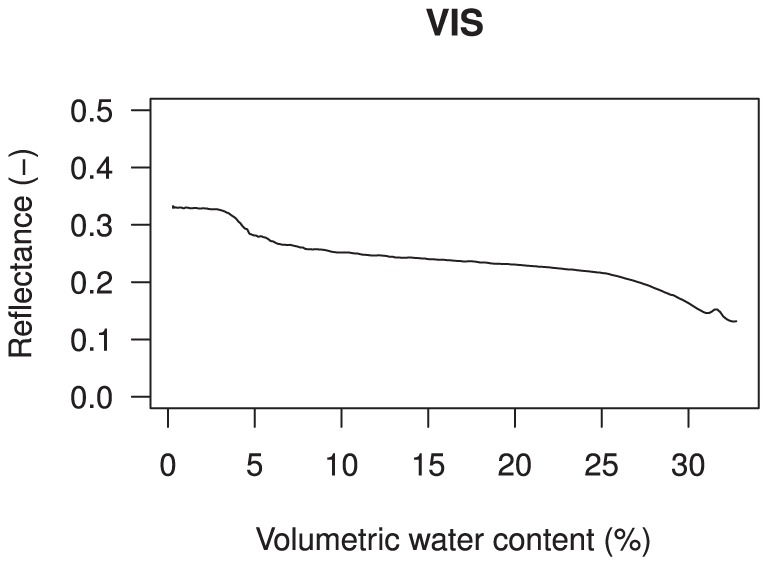
Averaged measured spectral reflectance upon wetting at visible wavelengths (400–700 nm), illustrating the limited contrast in reflectance between a dry and a wet beach composed of quartz sand.

It becomes clear from Figure 9 that, at visible wavelengths averaged over 400–700 nm, there is a limited contrast between dry and wet reflectance of beach sand composed of quartz sand. The slope of the reflectance curve is, after an initial steep decline, close to zero for intermediate moisture levels (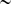
5–25%). This suggests that, for quartz sand beaches at visible wavelengths, only a distinction between a dry and a wet surface is practical. Differentiation of intermediate moisture levels would require very accurate radiometric calibration, also considering that other parameters affecting surface reflectance (e.g. mineral composition, grain size distribution, packing density, surface roughness) may vary under field conditions. This limited contrast is a plausible explanation for the weak correlation between beach surface moisture and reflectance found in the studies of [32]–[36] and [37]–[39]. A standard error of 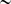 10% moisture content is reported by [40] and [41] for the photographic method, while with the terrestrial laser scanner (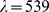 nm) the standard error increases after 7–8% moisture to such an extent that the method becomes impractical.

### Practical applications

The findings of our laboratory spectroscopy experiment imply that optical remote sensing of surface moisture is most effective when measuring in the near- and short-wave infrared. These wavelengths are strongly absorbed by water and provide sufficient contrast in the signal to differentiate intermediate moisture levels (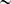
5–25%). The water absorption peaks of 1470 and 1940 nm are effective in particular. At these wavelengths there is enough contrast in the signal to also differentiate low moisture levels (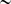
0–5%). Because of strong absorption by the atmosphere an active remote sensing technique is required, for example such as employed by [62] in a subsequent field experiment.

Ruessink et al. [62] tested the applicability of a RIEGL VZ-400 3D terrestrial laser scanner to derive spatially extensive moisture maps of a natural beach. The wavelength of this TLS is in the short-wave infrared at 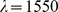 nm, hence on the wings of the absorption peak at 1470 nm. Ruessink et al. [62] deployed their scanner from a tripod at Egmond Beach in The Netherlands and collected 9 panorama scans (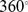 in the horizontal, 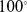 in the vertical, with 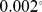 resolution in the horizontal and vertical). Each scan took about 10 minutes to complete and resulted in a cloud of 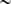
35,000,000 points. Simultaneously, 69 surface scrapings (thickness of a few millimetres) were taken, which were later on processed into gravimetric moisture estimates using standard laboratory techniques. The RIEGL VZ-400 outputs reflectance in decibels, with a 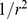 correction to account for the reduction of returned intensity with range 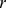. For 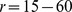 m [62] found a linear dependence between gravimetric surface moisture content and reflectance for the full range from dry to saturated sand. This confirms that the use of TLS with a wavelength near an absorption band is inherently more suitable to detect surface moisture over its full range than a TLS with a wavelength in the visible range (for example, [39]). The linear dependence is qualitatively consistent with Eq. (6), as decibel is a logarithmic unit. The standard error of their best-fit line amounted to about 2.6%, which is consiberably lower than reported for the photographic method of [32]–[36].

**Figure 10:**
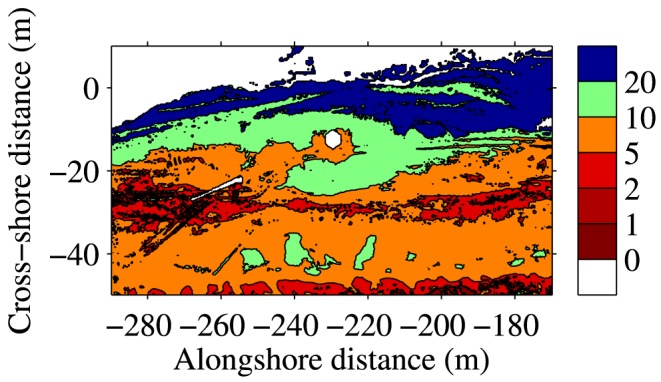
Example gravimetric moisture content (in %) map, estimated from the reflectance collected with a RIEGL VZ-400 3D terrestrial laser scanner at Egmond aan Zee, The Netherlands, from the upper dry beach (cross-shore distance 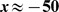 m) to the water line (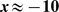 to 10 m) [62]. The local co-ordinates are positive in the seaward direction and to the south. The scanner was located at 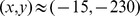 m. The slightly drier sand immediately around the scanner position is an artifact of the conversion from intensity to reflectance; close to the scanner, as also noted by [39], the imposed 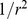 correction does not apply. The narrow bands with apparent lower moisture content (e.g. at alongshore 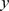 distances 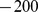 to 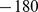 m and 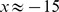 m) correspond to car tracks.

An example of a derived moisture map is provided in Figure 10. It illustrates the overall increase in moisture content from the dunefoot to the waterline, with superimposed variability related to secondary morphological highs and lows, consistent with [21]. The map also shows two narrow, approximately alongshore bands of apparent lower moisture content, which correspond to car tracks. While the sand in the car tracks may actually contain less pore water e.g. due to compaction, the collected reflectance could depend on surface roughness too. The rougher car tracks may increase the reflectance relative to a flat beach surface with the same moisture content. Nonetheless, [62]'s results illustrate the potential power of active remote sensing near a water absorption band to derive accurate surface moisture maps at a spatial and temporal resolution infeasible with other techniques. A future publication will describe and analyze Ruessink et al.'s [62] TLS data more extensively.

## Conclusions

In this study the effect of surface moisture content on spectral reflectance of coastal beach sand is measured and modeled for the the full optical domain of 350–2100 nm, to support practical applications for optical remote sensing of surface moisture. It is shown that:

- The effect of surface moisture on spectral reflectance of coastal beach sand can be described by an optical as well as a soil physical model.
- Near- and short-wave infrared wavelengths are most effective for relating surface moisture content to reflectance due to strong absorption of light in water.
- A terrestrial laser scanner operating at 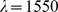 nm can derive accurate surface moisture maps at a high spatio-temporal resolution.
